# Effectiveness of Metformin Prolonged-Release Formulation on Achievement of Optimal Glycemic Control in Gestational Diabetes Mellitus: Protocol for a Pilot, Randomized, Double-Blind, Clinical Trial

**DOI:** 10.2196/79855

**Published:** 2025-12-19

**Authors:** Dittakarn Boriboonhirunsarn, Prasert Sunsaneevithayakul

**Affiliations:** 1 Department of Obstetrics and Gynecology Faculty of Medicine Siriraj Hospital Mahidol University Bangkok Thailand

**Keywords:** gestational diabetes mellitus, metformin, prolonged-release formulation, glycemic control, effectiveness

## Abstract

**Background:**

Gestational diabetes mellitus (GDM) is one of the most common complications in pregnancy. Optimal glycemic control is key to reducing the risk of adverse pregnancy outcomes. If glycemic control is inadequate, additional medications are needed. A large body of evidence has shown that metformin is an effective medication for GDM. Compared to the immediate-release formulation, the prolonged-release (PR) formulation of metformin offers some advantages with a single daily dose and has less frequent side effects, leading to better compliance. To date, no study has specifically reported the effectiveness of metformin PR in the treatment of GDM or the time required to achieve glycemic control after treatment. The results will help clinicians plan a better choice of treatment and follow-up for women with GDM and inadequate glycemic control.

**Objective:**

This study aims to evaluate the effectiveness of metformin PR in the treatment of GDM in terms of achieving glycemic control within 6 weeks, time to achieve glycemic control, and associated factors.

**Methods:**

A randomized, double-blind, placebo-controlled clinical trial will be conducted among 80 pregnant women diagnosed with GDM who had inadequate glycemic control at a university hospital in Thailand. The women will be randomized into 2 equal groups, receiving either metformin PR or a placebo in addition to nutritional therapy and behavioral modification. Dosage adjustment will be made every 2 weeks. If the glycemic target is not achieved within 6 weeks, insulin therapy will be initiated. All the participants and the investigators are blinded to the treatment provided. The primary outcome is the rate of achievement of glycemic control, and secondary outcomes are time to achieve glycemic control, rate of insulin therapy, and factors associated with the success of metformin PR use.

**Results:**

The study was funded in May 2025 when recruitment started. The study is projected to be completed in October 2026. Women with GDM who experienced inadequate glycemic control by nutritional therapy were assessed for eligibility. As of October 2025, 24 women with GDM agreed to participate and follow-ups were scheduled. The results are expected to be published in 2027.

**Conclusions:**

The results of this study will provide additional information on the use of metformin in the treatment of GDM, including the use of different formulations, rate of glycemic control, time to achieve glycemic control, and associated factors. This will help physicians better plan care for pregnant women with GDM, especially when glycemic control is inadequate, including choices of metformin formulation and dosage, follow-up schedule, and identification of women at risk of treatment failure.

**Trial Registration:**

Thai Clinical Trial Registry TCTR20250525007; https://www.thaiclinicaltrials.org/show/TCTR20250525007

**International Registered Report Identifier (IRRID):**

DERR1-10.2196/79855

## Introduction

Gestational diabetes mellitus (GDM) is one of the most common complications in pregnancy; it can lead to various adverse maternal and neonatal outcomes, including preeclampsia, cesarean section, large-for-gestational-age infants, macrosomia, birth injuries, shoulder dystocia, and neonatal hypoglycemia [1-3]. Optimal glycemic control (fasting plasma glucose [FPG] of <95 mg/dL and 2-h postprandial glucose, 2-h PPG of <120 mg/dL) is key to reducing the risk of developing adverse pregnancy outcomes [1-3].

In general, nutritional therapy, behavioral modification (including physical activity and exercise), and glucose monitoring and control are the main interventions to provide to all women with GDM to achieve glycemic targets. However, if glycemic control is inadequate, additional medications are needed. Insulin has long been used as the first-line medication in GDM. Nevertheless, a large body of evidence has consistently shown that metformin is also an effective medication for glycemic control in GDM. It has been reported that metformin helps in better glycemic control, better control of weight gain, reduction in adverse pregnancy outcomes, and reduction in insulin need and dosage requirement [4-14]. In addition, metformin has been reported to be safe to use during pregnancy [6,15-19] and appears to be more cost-effective compared to insulin in the treatment of GDM [20]. Currently, many international authorities recommend the use of metformin in women with GDM [1,21,22].

Different formulations of metformin are available, including an immediate-release (IR) formulation, with a time to maximum concentration of 3 to 4 hours and a 12-hour duration of action, and a prolonged-release (PR) formulation, with a time to maximum concentration of 7 to 8 hours and a 24-hour duration of action. Although both formulations have comparable effectiveness, metformin PR has advantages over its IR counterpart in terms of once-daily dosage with minimal gastrointestinal side effects, which helps improve compliance [23-28]. Gastrointestinal side effects have been reported with the use of metformin IR in previous studies, and some participants needed to stop the medication or reduce the dosage [4]. In another study, the use of metformin IR resulted in nausea and vomiting in 36% and 25% of the participants, respectively [5]. Therefore, metformin PR would be more suitable to use during pregnancy, not only because many pregnant women usually experience gastrointestinal symptoms, such as nausea and vomiting, but also because the medication has fewer side effects. Furthermore, with its once-daily dosage, compliance with the treatment will improve.

Although the benefits of metformin PR on diabetes have been reported, information on its potential benefits for the treatment of GDM is lacking. To address this knowledge gap, we will conduct a study to evaluate the effectiveness and associated characteristics of metformin PR in the treatment of GDM. The results will provide more insights into the use of metformin PR for the treatment of GDM. Understanding its effectiveness, time to achieve glycemic control, and the risk for treatment failure can help caring physicians make better decisions on treatment initiation, follow-up schedule for glycemic control evaluation, and identify those who are at higher risk for treatment failure. The results would be helpful in developing a higher quality of care for patients with GDM in the future.

Our hypothesis is that metformin PR is effective in GDM treatment in terms of the rate of adequate glycemic control and time to achieve glycemic target compared to nutritional therapy. Therefore, the primary objective of this study is to compare the rate of optimal glycemic control within 6 weeks between women with GDM who receive metformin PR and women who receive a placebo, in addition to standard nutritional therapy. The secondary objectives are to compare the time required to achieve optimal glycemic control and the rate of insulin therapy between the 2 groups, and to determine factors associated with the success of metformin PR treatment, such as prepregnancy BMI, degree of abnormalities of test results at the time of diagnosis, GDM risk factors, and other baseline characteristics.

## Methods

### Study Design

The study is designed as a randomized, double-blind, placebo-controlled trial and registered at the Thai Clinical Trial Registry (TCTR20250525007). Eligible participants will be randomly assigned in a 1:1 ratio to receive either metformin PR or a placebo. Each participant will be followed up every 2 weeks for glycemic control evaluation and dosage adjustment for 6 weeks.

According to the institutional practice guidelines, every woman with GDM will receive counseling by certified diabetes educator nurses and will be scheduled for follow-up every 2 weeks for glycemic control evaluation. If the glycemic target is not achieved after 6 weeks of intensive nutritional therapy, additional pharmacological treatment will be initiated. Therefore, this study will evaluate the primary outcome of glycemic control at 6 weeks after randomization.

### Study Setting

This study will be conducted at the Department of Obstetrics and Gynecology, Faculty of Medicine, Siriraj Hospital, Mahidol University, Bangkok, Thailand. Siriraj Hospital is the largest university-based tertiary care hospital in Thailand, with approximately 4000 deliveries per year. Routine GDM screening is offered to all pregnant women, and those with GDM are managed by a specialized care team, including an obstetrician, certified diabetes educator nurses, and endocrinologists. All women with GDM receive counseling regarding nutritional therapy, behavioral modification, and glycemic monitoring. Metformin has been incorporated into the guideline as an optional pharmacological therapy, as an alternative to insulin.

### GDM Diagnosis and Management

According to current institutional guidelines, GDM screening and diagnosis are offered to all pregnant women at the first antenatal care visit and repeated at 24 to 28 weeks of gestation if the initial screening results are normal. A 50-g glucose challenge test is used as a screening test with a 140 mg/dL cutoff value, and a 100-g oral glucose tolerance test is used for diagnosis using the Carpenter and Coustan criteria (≥2 abnormal values of 95, 180, 155, 140 mg/dL at 0, 1, 2, and 3 h). The current prevalence of GDM in the institution is approximately 15% to 20% of pregnant women.

After diagnosis, nutritional counseling and knowledge on behavioral modification are provided by certified diabetes educators. A glucometer is encouraged and offered for self-monitoring blood glucose (SMBG), but its use is based on women’s preference. FPG or 2-hour PPG, or both, are used for evaluation of glycemic control during follow-up. Glycemic targets are set at FPG <95 mg/dL and 2-hour PPG <120 mg/dL in >80% of the total SMBG test results.

If glycemic targets are not met at any time during pregnancy, repeat nutritional and behavioral counseling is offered. The women are reevaluated every 1 to 2 weeks. If adequate glycemic control is still not achieved within 4 weeks, pharmacological treatment will be initiated. In addition to insulin therapy, metformin will be offered as an alternative medication. The decision on choices of pharmacological therapy is based on the physician’s discretion and the woman’s preference.

### Eligibility Criteria

Eligible cases are pregnant women diagnosed with GDM who meet the inclusion and exclusion criteria, as described in Textbox 1.

**Inclusion and exclusion criteria.**

**Inclusion criteria**

- Aged ≥18 y
- Singleton pregnancy
- Diagnosed with gestational diabetes mellitus, according to institutional guidelines (50-g glucose challenge test followed by 100-g oral glucose tolerance test using the Carpenter and Coustan criteria)
- Do not achieve glycemic target after nutritional and behavioral counseling (fasting plasma glucose ≥95 mg/dL or 2-hour postprandial glucose ≥120 mg/dL in >20% of total self-monitoring blood glucose test results)

**Exclusion criteria**

- Definite or suspected pregestational diabetes mellitus
- Fetal congenital anomalies or deaths
- Suspected fetal growth restriction (as metformin is advised to be used with caution in those with fetal growth restriction)
- Currently using metformin or other antihyperglycemic treatment regardless of indication
- Contraindicated for metformin use

### Interventions

After initial screening and acquiring informed consent, the women will be randomly assigned to 2 groups—the intervention group or the control group.

For the intervention group, metformin PR will be provided to the participants, starting with 750 mg daily (1 tablet of metformin PR 750 mg). Follow-up is scheduled every 2 weeks for 6 weeks. Dosage adjustment will be made at each visit, depending on the results of glycemic control in a step-up protocol, that is, 750, 1000, 1500, and 2000 mg. The maximum dosage of metformin PR is 2000 mg per day. For dosage adjustment, 1 tablet of metformin PR 1000 mg will be used for a dosage of 1000 mg per day, 2 tablets of metformin PR 750 mg will be used for a dosage of 1500 mg per day, and 2 tablets of metformin PR 1000 mg will be used for a dosage of 2000 mg per day.

Pregnant women who are in the control group will receive 1 tablet of a placebo and will be scheduled for follow-up every 2 weeks for 6 weeks.

In addition to standard antenatal care according to institutional guidelines, pregnant women in both groups will receive individualized counseling on nutrition and behavioral modifications for glycemic control at every follow-up visit.

All participants are encouraged to adhere to the medication provided. A specific phone number will be provided to participants to receive an answer if they have any questions about the medications. Participants will be contacted every 2 days via a telephone call, SMS, or other online platforms to ensure medication adherence. Pill count will be performed at every visit, and additional encouragement will be provided. All participants are advised to avoid using other antidiabetic medications and other herbal products during the study period.

At the end of the study period of 6 weeks, glycemic control will be evaluated thoroughly, and appropriate treatment will be provided. Either the previous treatment will be continued or a new treatment will be initiated, after consultation with an endocrinologist, as appropriate. Standard antenatal care will also be provided until delivery. Treatment failure is defined as inadequate glycemic control after 2000 mg of metformin PR. In such a situation, insulin therapy is initiated.

If any of the conditions in the exclusion criteria are detected after enrollment, such as congenital anomalies, randomization concealment will be revealed and considered as a dropout, and these participants will not be included in the analysis. The pregnant woman will be thoroughly evaluated by the obstetrician team to provide appropriate management.

### Outcomes

The primary outcome of this study is the rate of achievement of optimal glycemic control within 6 weeks, which is defined as FPG <95 mg/dL or 2-hour PPG <120 mg/dL in >80% of total SMBG test results.

The secondary outcomes include time to achievement of optimal glycemic control, rate of insulin therapy, and rate of side effects. Treatment failure is defined as inadequate glycemic control after 2000 mg of metformin PR. In this situation, insulin therapy is initiated. Side effects will be evaluated by interviewing the participants at each visit and by additional contact via a telephone call, SMS, or other online platforms.

**Figure 1:**
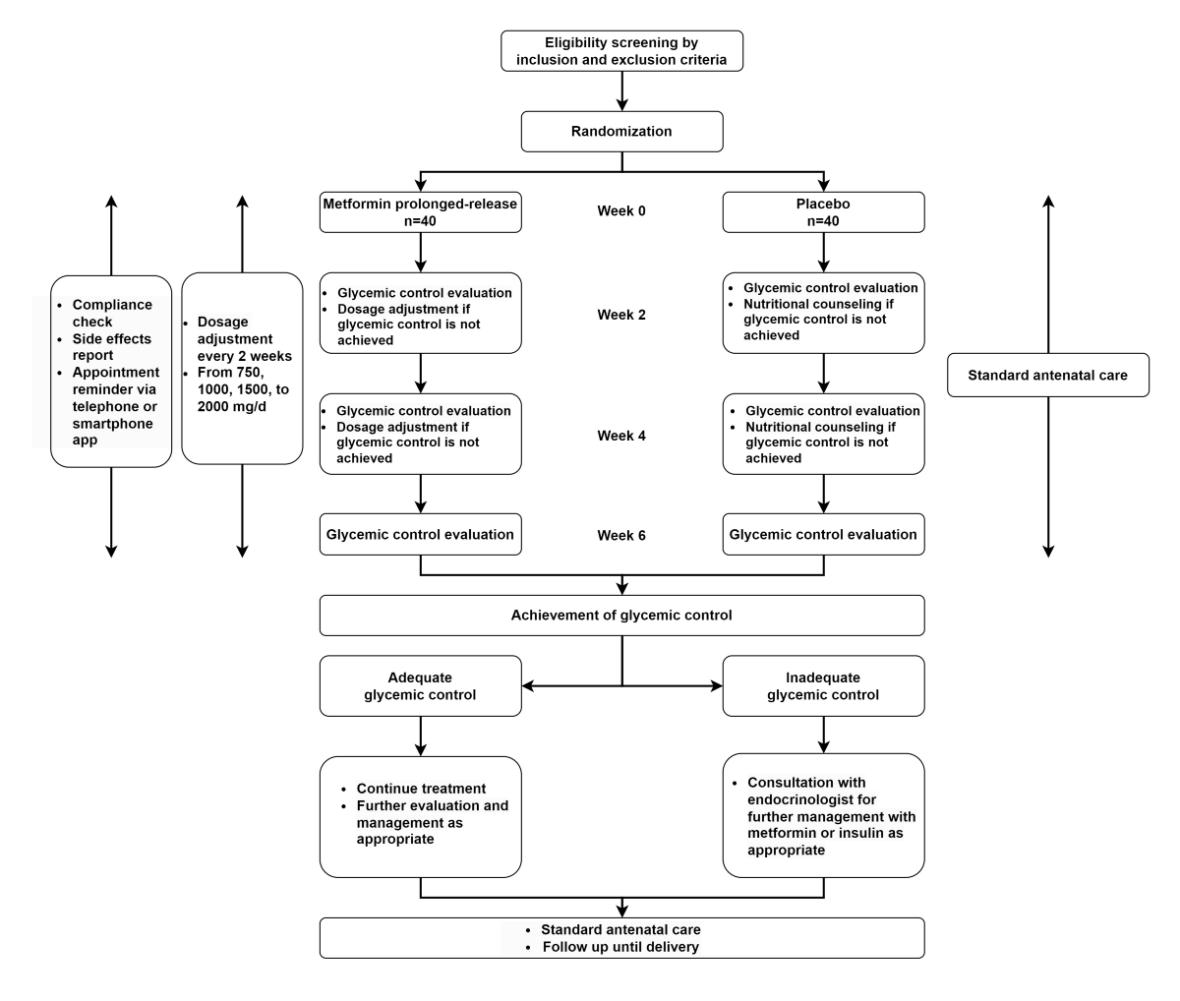
Study flow diagram.

The participant timeline is displayed in Figure 1.

The rate of achievement of glycemic target within 6 weeks among women with GDM receiving metformin PR and placebo is estimated to be 95% and 70%, respectively, from a pilot data collection. At 95% confidence and 80% power, at least 40 women with GDM are required in each group, estimating a loss of 20% of the participants.

### Assignment of Interventions

#### Randomization

Participants will be allocated to receive either metformin PR or a placebo by simple randomization. Central randomization will be used to minimize the risk of bias. The investigator will contact an independent individual to obtain the randomization list at the time of enrollment of each participant.

#### Blinding

All participants will be blinded to the treatment received. Placebos will be prepared by a pharmacist to resemble metformin PR in shape, size, and color. All caring obstetricians will be blinded to the treatment and make decisions on dosage adjustment or initiation of insulin therapy at their discretion. Glycemic control will be blindly assessed by the investigators without knowing the group to which the participants are assigned.

In case of withdrawal or termination of the study protocol, such as the occurrence of severe adverse events or noncompliance, the treatment will be unblinded. Further individualized management will be provided as appropriate. In addition, emergency unblinding will be performed if the participants need to be evaluated or treated for medical or obstetric conditions other than GDM.

### Data Collection and Management

Data will be collected at enrollment and at every visit. Data will be entered into the electronic case record form, including baseline and obstetric characteristics, such as age, parity, and prepregnancy BMI. GDM diagnosis and management data, such as gestational age at diagnosis, results of the 50-g glucose challenge test and 100-g oral glucose tolerance test, and glycated hemoglobin, will be collected. Glycemic control data, including FPG and 2-hour PPG at every visit, or percentage of abnormal SMBG test results, will also be noted. Furthermore, the time to achieve the glycemic target will be evaluated, as will side effects, such as bloating, nausea, and vomiting.

Data at every time point will be checked for completeness and entered into the electronic case record form using an electronic data capture system. Data range and values will be rechecked by another research assistant. Incomplete and invalid data will be rechecked with those in the case record form or medical record. An audit trail will be used to track and identify all actions performed on the data, ensuring data integrity.

### Statistical Methods

Descriptive statistics, including number, percentage, mean, and SD, will be used to describe variables as appropriate. Comparisons of variables between groups will be made using the 2-tailed Student *t* test for continuous variables, such as age, BMI, and gestational age, or the chi-square test for categorical variables, such as parity and GDM risk factors. Comparison of rates of achievement of optimal glycemic control between groups will be performed using the chi-square test. Relative risks and 95% CIs will be evaluated. Time to achieve glycemic control will be compared between groups, using the Student *t* test or Mann-Whitney *U* test, depending on the distribution of the variables. Kaplan Meier curves will be used to estimate the survival curve of time to achieve glycemic control after treatment, and the log-rank test will be used to compare the survival curves between treatment and control groups. Rates of insulin therapy and side effects will be compared using appropriate statistical tests. Missing values will be handled using multiple imputation methods, as appropriate. Subgroup analysis by BMI categories, parity, or GDM risks will be performed as appropriate. All analyses will be performed on an intention-to-treat basis. A *P* value of <.05 is considered to indicate statistical significance.

### Oversight and Monitoring

#### Safety Management

All participants will receive standard antenatal care at every visit by obstetricians. In addition to physical examination, obstetric examination will be performed, including evaluation of fetal growth and fetal heart rate activity, either by clinical or ultrasonographic examinations. Additional procedures will be provided as appropriate, such as electronic fetal monitoring, detailed ultrasonographic examination, etc.

Glycemic control will be evaluated by FPG and 2-hour PPG or percentage of abnormal SMBG test results. Individual nutritional and behavioral counseling will be provided, customized for each participant. According to institutional guidelines, participants with FPG ≥200 mg/dL or glycated hemoglobin ≥10% will be admitted for further evaluation and initiation of insulin therapy by an endocrinologist.

#### Adverse Event Reporting and Harms

Any adverse events will be evaluated by self-report from the participants and by interview and clinical evaluation during each visit (every 2 weeks), starting from the day of enrollment until the end of the study (at 6 weeks after enrollment). In addition, participants will be contacted every 2 days via a telephone call, SMS, or other online platform to evaluate any adverse events. A telephone number will be given to all participants to call if there are any suspected abnormalities or any adverse events. Attending obstetricians will assess whether the events are related to the investigational drugs used for GDM. Management will be provided as appropriate by attending obstetricians, and consultation with other specialists will be made as necessary. In case of any adverse events, an additional specific follow-up schedule with related specialists will be offered until they are resolved or considered stable. Assessment of the adverse events and their response to treatment will be by telephone call, self-report, and clinical and laboratory assessment, as appropriate, according to the contexts and nature of the events. All the events will be followed up on until the conditions resolve. All the adverse events will be recorded in detail in the case record form.

In case of withdrawal or termination of the study protocol, such as severe adverse events or noncompliance, treatment will be unblinded. Further individualized management will be provided as appropriate.

#### Data Safety Monitoring Board

An independent data safety monitoring board will be set up for trial monitoring. The members will include 2 obstetricians, 1 endocrinologist, 1 biostatistician, and 1 methodology expert. None of the data safety monitoring board members has any conflicts of interest that may affect the decision-making process.

### Ethical Considerations

At the antenatal care clinic, the investigator or the research assistant will screen pregnant women with GDM for eligibility and provide detailed information regarding the study. If the women agree to participate, a written informed consent will be obtained. Each participant will receive compensation of US $30. The study has been approved by the Siriraj Institutional Review Board (Si106/2025). All personal identification information of the participants, including names, telephone numbers, hospital numbers, and medical records, will be anonymized to ensure confidentiality. Coding will be used for each participant, and no related information will be disclosed. All study data will be stored by the researchers and will be retained for at least 5 years after publication.

### Dissemination

The research findings will be disseminated to relevant stakeholders, including participants, their families, physicians, and the institutional medical community. In addition, the results will be disseminated via high-impact international peer-reviewed medical journals and presentations at international medical conferences to ensure broad academic and clinical engagement. Reporting the results of the study will follow the latest CONSORT (Consolidated Standards of Reporting Trials) guidelines. Distribution of key information will also be made available for health care professionals and the public, as appropriate.

The findings will also be disseminated at the national level via various medical institutions, obstetrics and gynecology communities, endocrinology communities, and the Ministry of Public Health. These findings could help modify the current guidelines for GDM management.

## Results

The study was funded in May 2025, when recruitment started. Women with GDM who had inadequate glycemic control by nutritional therapy were assessed for eligibility. As of June 2025, 24 women with GDM agreed to participate and follow-ups were scheduled. Recruitment will continue until 80 participants are included, which is expected to occur by September 2026. The study is projected to be completed in October 2026. The results are expected to be published in 2027.

## Discussion

### Anticipated Findings and Importance

Although the effectiveness of metformin in the treatment of GDM has been demonstrated in previous studies, only metformin IR has been investigated [4-14]. No study has specifically reported the effectiveness of metformin PR, which could have advantages over metformin IR in terms of fewer side effects and a once-daily dosage that results in better compliance [23-28]. Moreover, no previous study has reported the time required to achieve glycemic control after metformin treatment.

Therefore, the results of this study will add additional information to the medical community. It can help caring physicians to make better decisions regarding the selection of appropriate dosage and formulation of metformin, as well as a follow-up schedule for evaluation of glycemic control. In addition, the results could also help identify women at a higher risk for unsuccessful glycemic control after metformin therapy.

Some challenges in conducting the study might include adherence to the scheduled treatment and follow-up. Various measures are planned to improve both important issues. All participants will be informed regarding the importance of adherence to the treatment and follow-up to achieve glycemic control. Participants will be regularly contacted every 2 days via a telephone call, SMS, or other online platforms to encourage adherence to the provided medications and follow-up schedule, and for the evaluation of any adverse events.

The results will provide more insights into the use of metformin PR for the treatment of GDM. Understanding its effectiveness, time to achieve glycemic control, as well as the risk for treatment failure, can help caring physicians make better decisions on treatment initiation and follow-up schedule for glycemic control evaluation, and identify those who are at higher risk for treatment failure. All the information would be helpful in developing a higher quality of care for patients with GDM in the future.

### Strengths and Limitations

The strengths of this study include its randomized, double-blind, placebo-controlled trial design. The diagnosis of GDM follows a uniform institutional guideline. Nutritional and behavioral modifications counseling is provided by well-trained, certified diabetes educator nurses. The main outcome of glycemic control is objectively measured.

Some limitations include that the duration of the intervention and follow-up to evaluate the primary outcome of glycemic control within 6 weeks might not fully capture the long-term effects or improvements after metformin PR treatment. A longer follow-up period is needed to determine whether a higher dose of metformin PR or insulin will be required to achieve adequate glycemic control as pregnancy progresses. In addition, the actual dietary and behavioral practice varies among participants and cannot be accurately measured, which could affect the outcomes. Moreover, the study does not provide a head-to-head comparison between different formulations of metformin, that is, IR versus PR.

The study is not primarily designed to determine if the use of metformin PR improves the pregnancy outcomes, as the study ends at 6 weeks after initiation of the treatment, and further management will vary according to the status of the women and physicians’ discretion. Further well-designed studies on the improvement of GDM-related maternal and neonatal outcomes, such as preeclampsia, large-for-gestational-age infants, and macrosomia, with metformin therapy should be conducted to provide additional evidence on metformin benefits and help aid clinicians’ decisions.

The study is planned to be a pilot randomized clinical trial, and the sample size was calculated based only on the primary objective of comparing glycemic control between the 2 groups and did not take into account for some related factors, such as BMI or gestational age. However, this issue should be considered when designing larger definitive studies in the future. Acknowledging these limitations is essential for interpreting the results and refining future research.

### Conclusions

This study is primarily aimed at investigating whether metformin PR is effective in the treatment of GDM in terms of achievement of glycemic control within 6 weeks and time to achieve glycemic control. This information will provide additional evidence on the potential benefits of metformin treatment in GDM that can be applied to clinical practice. If metformin PR is effective in achieving glycemic control within a short time, it can be considered a good alternative to conventional IR formulation or insulin. In addition, the results will serve as a basis for future research to explore the benefits of treatment, especially if the treatment can improve maternal and neonatal outcomes.

## Data Availability

The datasets generated or analyzed during this study are available from the corresponding author on reasonable request.

## Abbreviations

FPG: fasting plasma glucose
GDM: gestational diabetes mellitus
IR: immediate-release
PR: prolonged-release
SMBG: self-monitoring blood glucose
